# Cell-free adipose extract inhibits hypertrophic scar formation through collagen remodeling and antiangiogenesis

**DOI:** 10.1515/med-2025-1249

**Published:** 2025-08-22

**Authors:** Junchao Sun, Yujie Zhao, Zhoujiang Qu, Shudong Sun, Kun Wang

**Affiliations:** School of Clinical Medicine, Shandong Second Medical University, Weifang, China; Department of Burns and Wound Repair, Weifang People’s Hospital, Shandong Second Medical University, Weifang, China

**Keywords:** hypertrophic scar, cell-free adipose extract, CEFAE, collagen, angiogenesis, antiangiogenesis

## Abstract

**Objective:**

Hypertrophic scars (HS) are a fibrotic proliferative disorder that results from an abnormal wound healing process, presenting significant challenges for clinical intervention. The primary characteristics of HS include excessive collagen deposition and angiogenesis. In recent years, the study of mesenchymal stem cells (MSCs) and their derived exosomes has emerged as a prominent area of research within the academic community. However, the therapeutic application of MSCs is impeded by several challenges, including immune rejection, sourcing limitations, ethical dilemmas, and difficulties related to the scalability of exosome production. Cell-free adipose extract (CEFAE), a novel bioproduct derived from adipose tissue, is rich in various active protein factors that are essential for MSCs and their exosomes. CEFAE presents several advantages, including low immunogenicity, non-tumorigenicity, and a high degree of clinical safety. However, the application of CEFAE in the prevention and treatment of scar formation has not been adequately validated through experimental studies.

**Methods:**

This research established a rabbit ear scar model, establishing a control group, a low-concentration CEFAE group (L-CEFAE), and a high-concentration CEFAE group (H-CEFAE) to evaluate wound treatment. Observations of scar changes were conducted at 14 and 28 days post-treatment, supplemented by histological and immunohistochemical analyses.

**Results:**

Histological analysis revealed that the H-CEFAE group achieved optimal outcomes, with the lowest collagen deposition, thinnest epidermal/dermal thickness, and the most orderly collagen alignment. Furthermore, the formation of new blood vessels in the H-CEFAE group showed a significant reduction over time, resulting in decreased blood supply, which is beneficial for suppressing scar tissue development. Quantification of COL I, COL III, and vascular endothelial growth factor also supports these results.

**Conclusion:**

The findings indicated that high-concentration CEFAE has a beneficial preventive and therapeutic effect on scar proliferation. Furthermore, the study explored the potential mechanisms by which CEFAE inhibits scar proliferation, thereby providing novel therapeutic strategies for the prevention and management of clinical scars.

## 1 Introduction

Hypertrophic scars (HS) typically develop following severe burns or skin injuries and are classified as a fibrotic proliferative disorder that occurs during the skin wound healing process [1,2,3,4]. These scars are characterized by excessive proliferation of fibroblasts, fibrosis, chronic inflammation, and an overabundance of extracellular matrix (ECM) proteins produced by fibroblasts, particularly type I and type III collagen [5,6,7,8,9,10]. HS not only cause cosmetic concerns but may also lead to functional impairments, resulting in both physiological and psychological issues. Currently, there are various treatment methods for scars in clinical practice, commonly including surgical excision, laser therapy, pressure therapy, local injection of corticosteroids, and functional dressings [11,12,13,14,15]. Since there has not been an effective treatment plan that can completely resolve the issue of scars, there is an urgent need to explore new clinical intervention approaches.

In recent years, mesenchymal stem cells (MSCs) and their derived exosomes have emerged as a prominent area of research in academia [16,17,18,19,20]. For instance, exosomes derived from human adipose-derived MSCs have been utilized to mitigate HS fibrosis [21]. Nevertheless, MSC therapy continues to encounter numerous unresolved challenges, including immune rejection, sourcing limitations, ethical controversies, and difficulties in scaling up exosome production [17,22,23]. These factors collectively hinder the advancement of stem cells and exosomes in clinical applications. Research has demonstrated that stem cells can facilitate tissue repair by secreting growth factors [21,24,25,26,27,28]. However, the low yield of cytokines and the high cost of treatment present significant barriers to their widespread application.

Fat tissue has been extensively utilized in the treatment of various diseases after undergoing processing through physical or chemical methods as they are rich in cytokines [29,30,31,32]. Cell-free adipose extract (CEFAE) is an emerging biological product derived from adipose tissue that has garnered attention in regenerative medicine. Unlike traditional fat grafting or stem cell therapies, CEFAE is processed to remove intact cells (e.g., adipocytes, stromal vascular fraction cells) while retaining a concentrated mixture of bioactive components, including growth factors [e.g., vascular endothelial growth factor (VEGF), FGF, TGF-β], cytokines, extracellular vesicles, and other soluble proteins [33,34,35,36,37,38]. The extract is typically prepared through mechanical emulsification, centrifugation, and filtration of lipoaspirated fat, resulting in a liquid formulation enriched with regenerative molecules but devoid of cellular debris or risks associated with whole-cell transplantation. Furthermore, CEFAE offers several advantages, including autologous origin, low immunogenicity, non-tumorigenicity, high clinical safety, and minimal ethical concerns [39,40,41,42,43,44]. However, the application of CEFAE in the prevention and treatment of scar formation lacks robust experimental validation. This study employs a rabbit ear scar model to investigate the preventive and therapeutic effects of CEFAE on scar hyperplasia and further explores its potential mechanisms for inhibiting scar hyperplasia, thereby providing new treatment strategies for the prevention and management of clinical scars.

## 2 Materials and methods

### 2.1 Preparation of CEFAE

A total of 24 male New Zealand rabbits aged 8–10 weeks were used in this study and purchased from Qingdao Kangda Aibo Biotechnology Co., Ltd. (Qingdao, China). The animals were housed under controlled conditions (24 ± 2°C, 50 ± 10% humidity, 12 h light/dark cycle) and acclimatized for 1 week. All experiments and procedures were approved by the Animal Research Committee of the Clinical Medical College of Shandong Second Medical University.

Using negative pressure suction technology, 5 mL of adipose tissue was collected from the groin area of a rabbit. The collected adipose tissue was rinsed with physiological saline and then centrifuged at 1,200 rpm for 3 min. The excess washing liquid from the lower layer was drained, and the above steps were repeated for a total of three cycles. After centrifugation, the adipose tissue was divided into 10 mL syringes using a fat emulsifier. One end of the emulsifier was connected to a 10 mL syringe, while the other end was connected to an empty 10 mL syringe. After manually pushing and pulling the fat 60 times, the emulsified fat was transferred to a new syringe, the stopper was tightened, and the syringe piston shaft was broken off. The remaining fat was processed in the same manner until all the fat was handled. During the emulsification process, if larger fat clumps or obvious white fascia were observed, they could be removed and discarded using sterile gauze or tweezers. After balancing the emulsified fat, it was symmetrically placed in a centrifuge and centrifuged at 2,000 rpm for 10 min. Following centrifugation, a three-layer structure should form, with the bottom layer being CEFAE, the middle layer being fat, and the top layer being oil. The top two layers were removed to obtain pure CEFAE. Finally, the obtained CEFAE was filtered using a 0.22 micron sterilizing filter, and the filtrate was aliquoted into 1 mL sterile tubes for future use.

### 2.2 Establishment and treatment of rabbit ear scar model

The experimental rabbits were anesthetized using inhalation of 2–2.5% isoflurane. Once adequate anesthesia was confirmed, three circular defects, each with a diameter of 8 mm, were created on the inner surface of the ventral side of each ear, removing the dermis, epidermis, and perichondrium.

These three defects were randomly assigned to three groups: the top group received high concentration CEFAE with 3 mg/mL (H-CEFAE group) treatment, the middle group received low concentration CEFAE with 1 mg/mL (L-CEFAE group) treatment, and the bottom group served as the PBS control group. At a distance of 1 mm from the outer edge of the wound, each group received a subcutaneous injection of H-CEFAE, L-CEFAE, or PBS, with an injection volume of 100 µL for each.

After a 14-day observation period, twelve experimental rabbits (four in each group) were anesthetized using 2–2.5% isoflurane. Subsequently, sterile ophthalmic scissors were employed to incise the muscle layer, allowing for the excision of the wound and the surrounding tissue within a 3 mm margin. The fresh wound tissue was immediately fixed in 4% formaldehyde for subsequent dehydration and paraffin block preparation. After 28 days, the same procedure was conducted on the remaining twelve rabbits.

### 2.3 Scar elevation index (SEI)

The SEI is utilized to evaluate the condition of scars and the process of wound healing. This index is determined by measuring the distance from the highest point of the vertical scar tissue (a) to the surface of the surrounding normal skin (b) and to the surface of the ear cartilage. The specific methodology involves using ImageJ software for measurement, where SEI is calculated as SEI = *a*/*b*. In this formula, ‘*a*’ represents the vertical distance from the highest point of the scar tissue to the surface of the ear cartilage, while ‘*b*’ denotes the vertical distance from the surface of the surrounding normal skin to the surface of the ear cartilage. The assessment of the SEI is performed prior to injection and again at 14 and 28 days post-injection.

### 2.4 Histopathological examination

Fix the samples in 4% paraformaldehyde, followed by embedding in paraffin. Then, cut the samples into sections with a thickness of 5 µm and mount them on glass slides. The slices are processed through the following steps: first, they undergo routine deparaffinization and hydration treatment, then they are soaked in hematoxylin for 5 min, followed by rinsing with tap water. Next, the slices are immersed in a hydrochloric acid-ethanol solution for 30 s, rinsed again with tap water, and then stained with eosin for 2 min, followed by another rinse with tap water. After that, a gradient concentration ethanol dehydration treatment is performed, and finally, they are cleaned in xylene and mounted with neutral balsam. The final results are observed under a microscope.

The tissue sections were subjected to staining with Ponceau acid fuchsin for a duration of 10 min, followed by rinsing with distilled water. Subsequently, the sections were immersed in a phosphomolybdic acid solution for 5 min, after which they were stained with aniline blue for an additional 5 min and treated with 1% acetic acid for 2 min. Following these procedures, the sections underwent dehydration, clarification, and mounting before being examined under a microscope.

### 2.5 Immunohistochemistry and Immunofluorescence staining

Fix the samples in 4% paraformaldehyde, followed by embedding in paraffin. Then, cut the samples into sections with a thickness of 5 µm and mount them on glass slides. After deparaffinization with xylene, the sections underwent gradient alcohol hydration and were heated in a microwave to promote antigen retrieval. Next, the sections were incubated in a 3% H_2_O_2_ solution for 15 min to eliminate endogenous peroxidase activity. Subsequently, the sections were blocked with goat serum for 1 h to prevent non-specific binding. The sections were incubated overnight with the primary antibody at 4°C, followed by a 2 h incubation with the secondary antibody. The color development process used diaminobenzidine. After counterstaining with hematoxylin, the sections were dehydrated and cleared, and finally sealed with resin.

Tissue sections are placed in a repair box filled with EDTA antigen retrieval buffer (pH 9.0) and subjected to antigen retrieval in a microwave. Heat on high for 3 min until boiling, then switch to low for 9 min. After natural cooling, the slides are washed once in PBS for 5 min. After gently shaking off excess liquid, 10% rabbit serum is added to evenly cover the tissue, and the slides are incubated in a 37°C oven for 30 min. The blocking solution is gently discarded, and PBS with a prepared primary antibody is added to the slides, which are then placed in a humid box and incubated overnight at 4°C. The slides are washed in PBS three times, with each wash lasting 5 min. After gently shaking off excess liquid, a secondary antibody is added to cover the tissue and incubated at 37°C for 45 min in the dark. The slides are washed in PBS four times, with each wash lasting 5 min, and after gently shaking off excess liquid, DAPI staining solution is added and incubated at room temperature for 10 min in the dark. The slides are washed in distilled water three times, with each wash lasting 5 min. After gently shaking off excess liquid, the slides are mounted with an anti-fade mounting medium. The slides are observed under an inverted fluorescence microscope, and images are collected. The results were analyzed using ImageJ software.

### 2.6 Statistical analysis

Differences in quantitative data were analyzed using SPSS 23. A *p*-value of less than 0.05 was considered statistically significant.


**Ethical approval:** The study protocol was reviewed and approved by the Shandong Second Medical University Research Ethics Committee and Weifang People’s Hospital Research Ethics Committee.

## 3 Results

### 3.1 *In vivo* treatment

**Figure 1:**
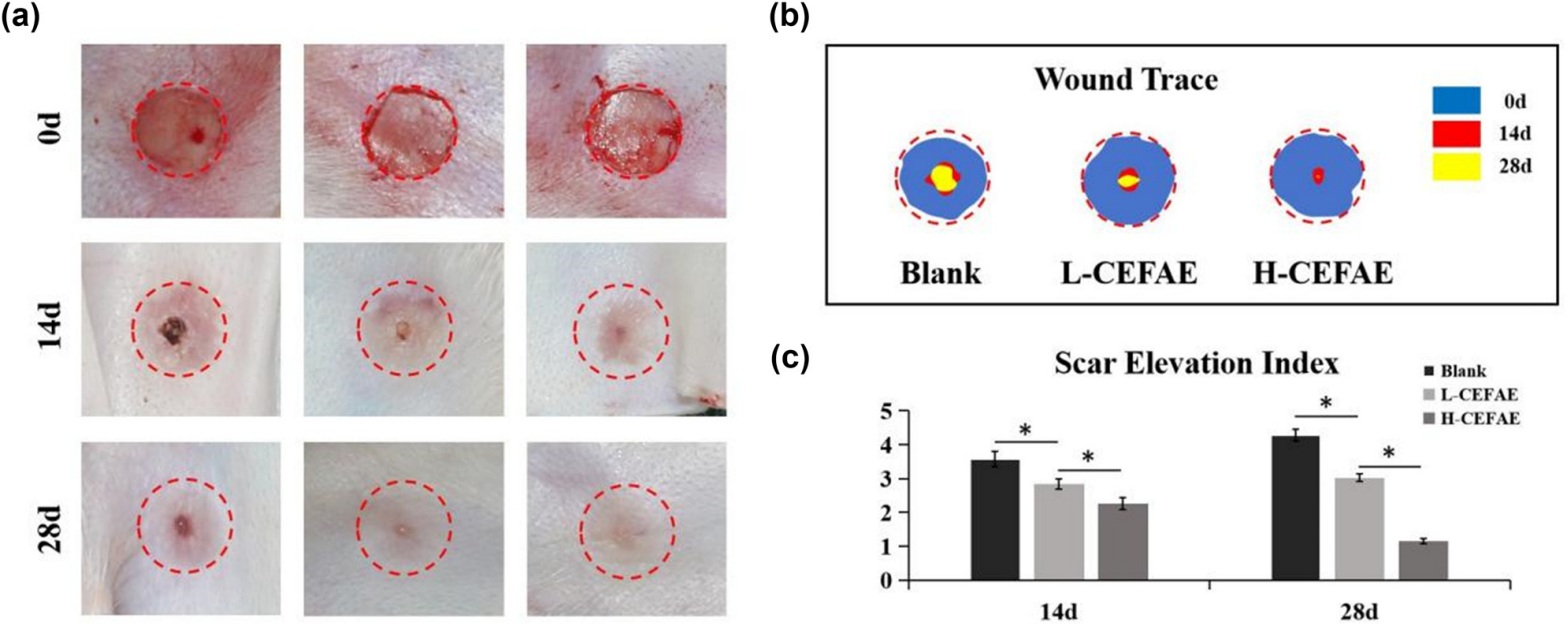
Treatment of HS: (a) Gross view, (b) Wound trance of Blank, L-CEFAE, and H-CEFAE group at 0, 14, and 28 days; (c) SEI assay calculated from HE staining (*P < 0.05).

Before starting the treatment, all rabbits were successfully modeled, and three circular wounds with a diameter of 0.8 cm were made on each rabbit's ear, and the cartilage membrane was removed. Comprehensive observations indicated that by the 14th day following surgery, the wound healing rates in both the H-CEFAE and L-CEFAE groups surpassed those of the Blank group. By the 28th day, all groups achieved complete wound closure; however, the Blank and L-CEFAE groups exhibited notable scar characteristics, including elevation, contraction, erythema, and palpable scars. In contrast, the H-CEFAE group presented with flatter scars, demonstrating less contraction and reduced erythema (Figure 1). A quantitative measure of the SEI was significantly lower in the H-CEFAE group (1.15 ± 0.08) compared to the L-CEFAE (3.03 ± 0.12) and blank (4.28 ± 0.17) groups at day 28. These findings suggest that the CEFAE effectively reduces scar hypertrophy and promotes regenerative healing.

### 3.2 Histopathological, immunohistochemistry and ommunofluorescence staining

**Figure 2:**
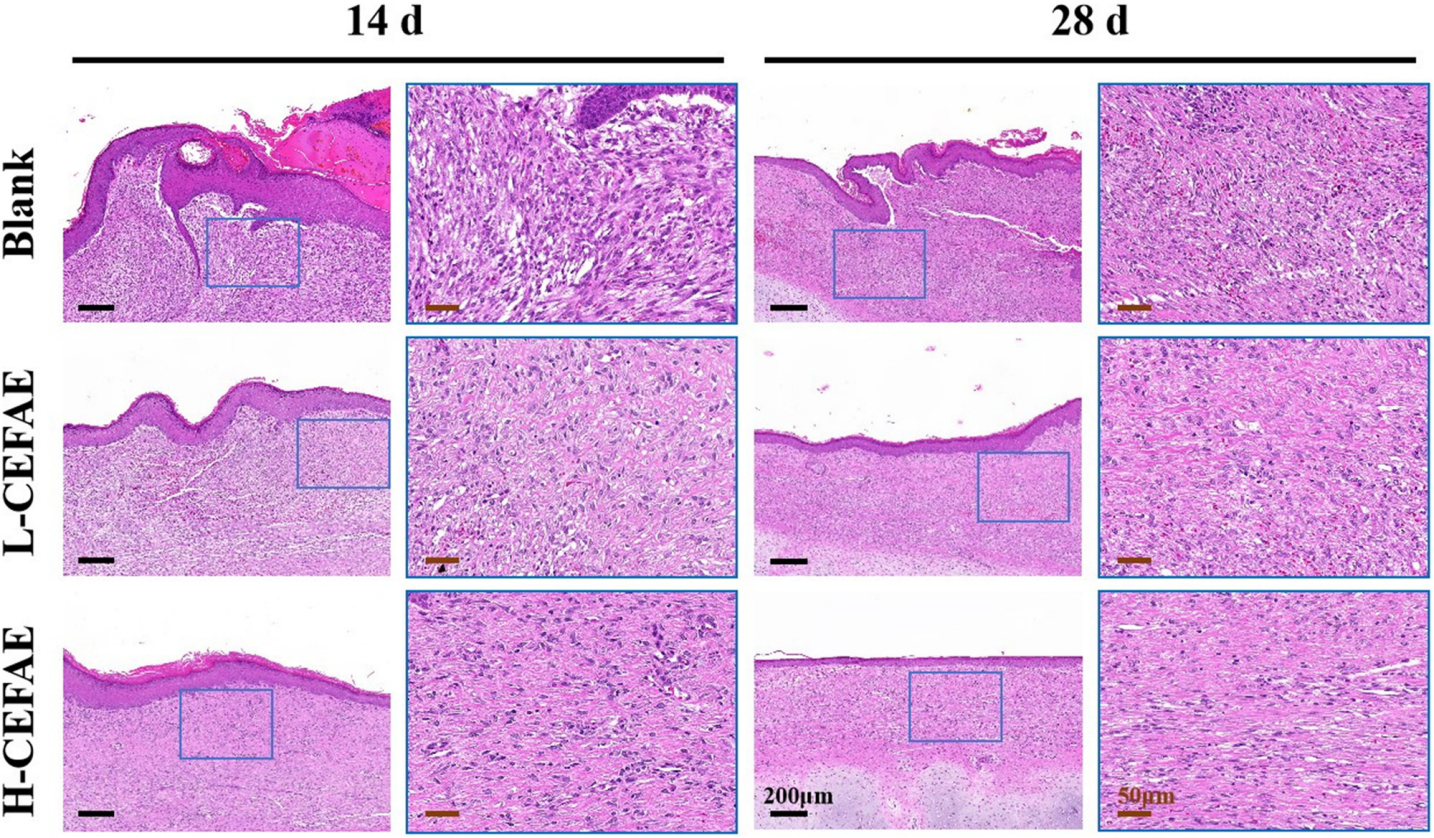
HE staining of blank, L-CEFAE and H-CEFAE with 14 and 28 days.

**Figure 3:**
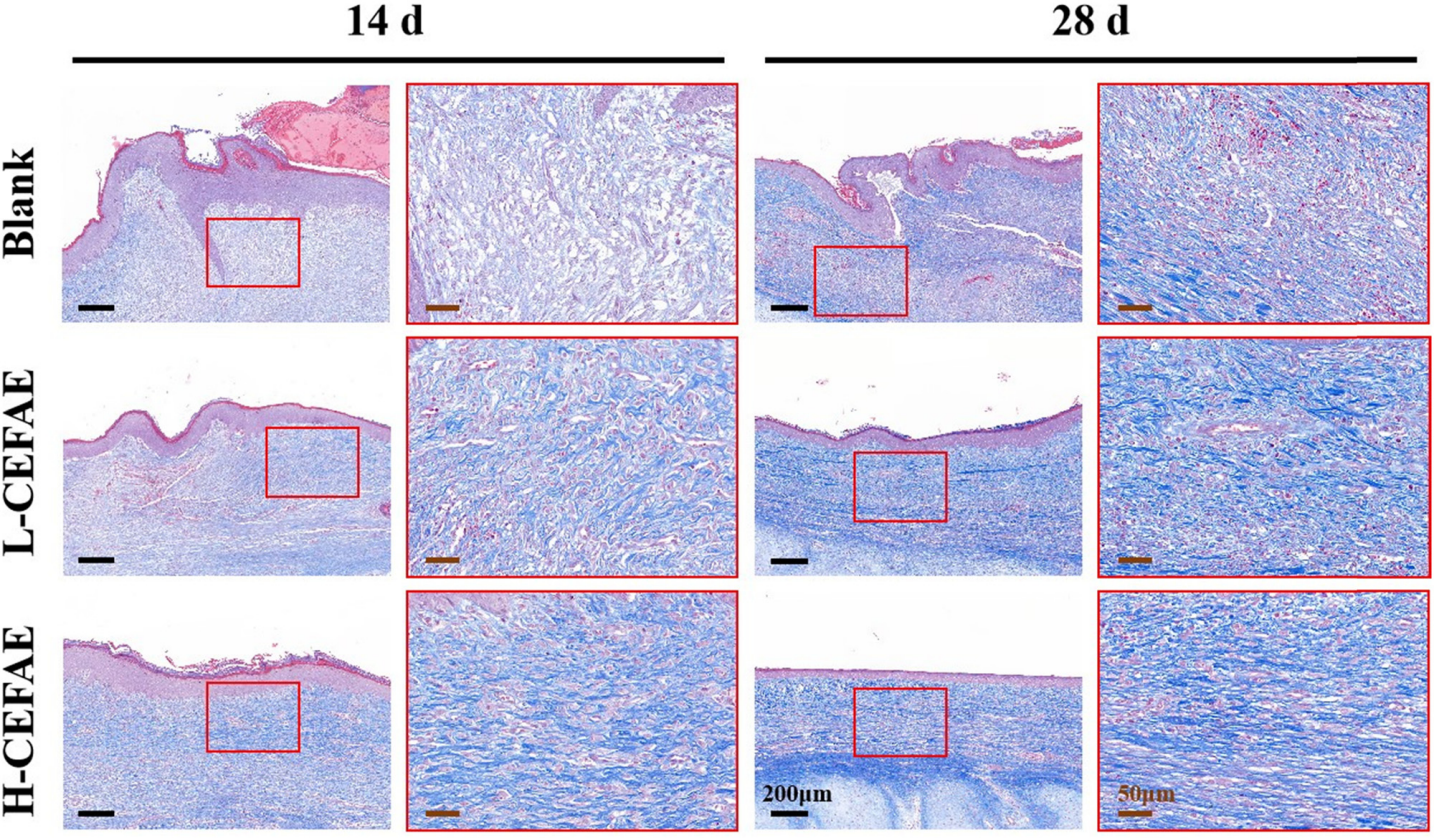
Masson staining of blank, L-CEFAE, and H-CEFAE at 14 and 28 days.

**Figure 4:**
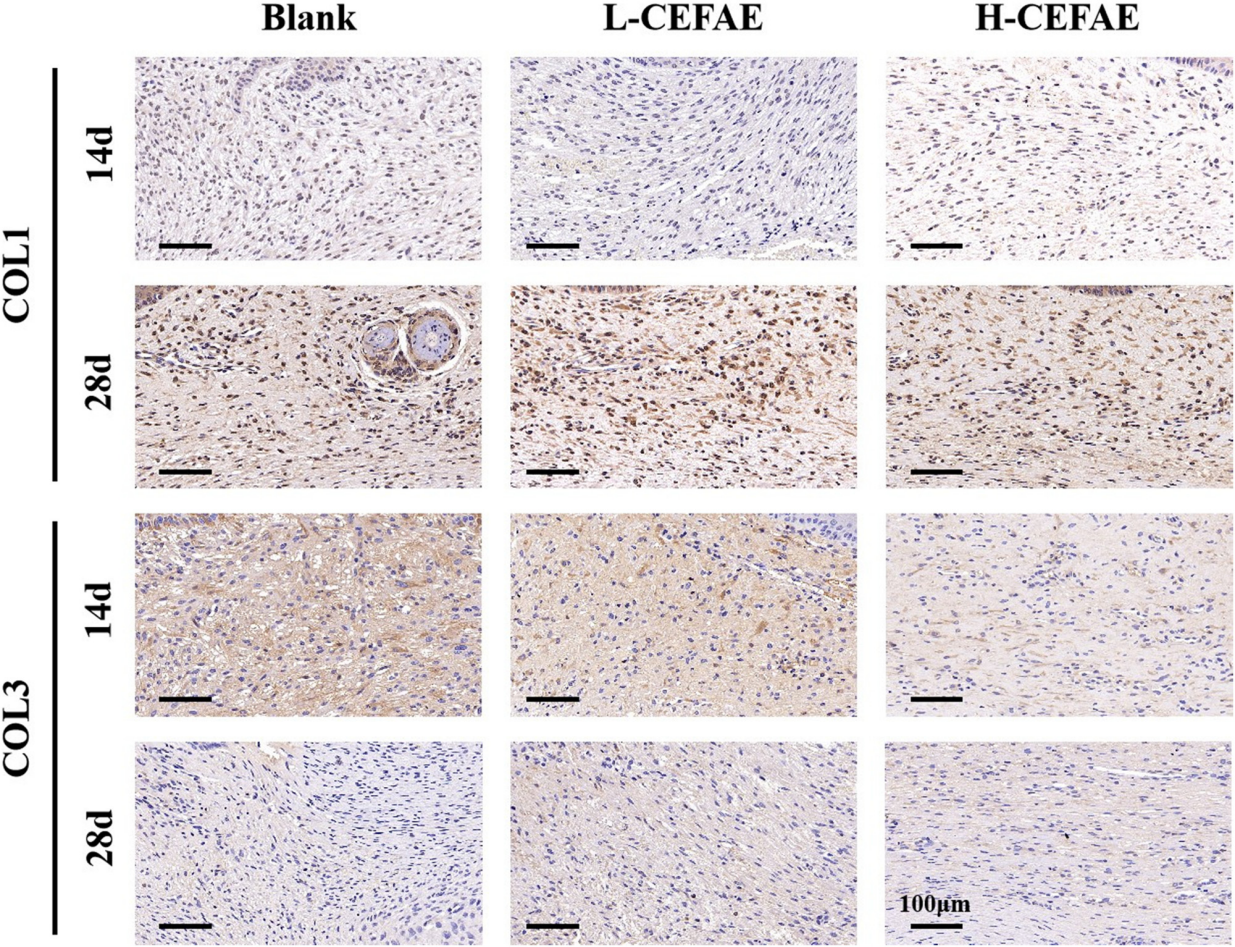
COL I and COL III staining of blank, L-CEFAE, and H-CEFAE at 14 and 28 days.

**Figure 5:**
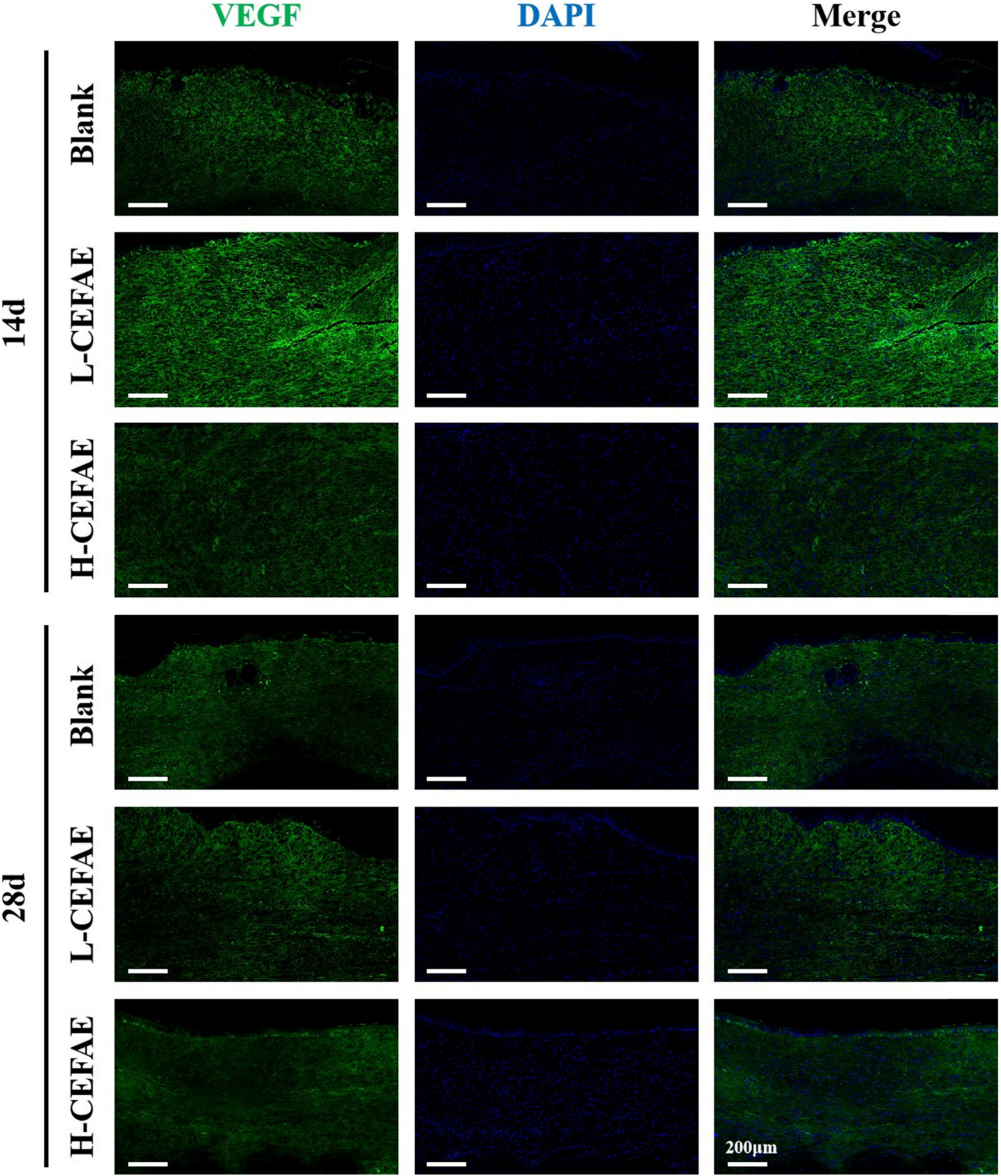
VEGF staining of blank, L-CEFAE, and H-CEFAE at 14 and 28 days.

**Figure 6:**
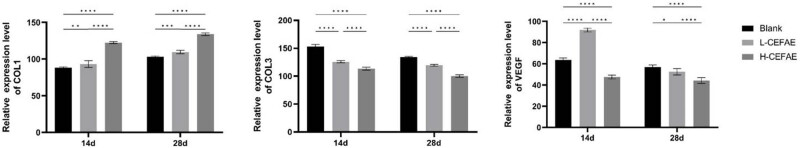
Quantification of COL I, COL III, and VEGF.

Histological analysis revealed that the H-CEFAE group achieved optimal outcomes, with the lowest collagen deposition, thinnest epidermal/dermal thickness, and the most orderly collagen alignment. The L-CEFAE group showed intermediate efficacy, demonstrating comparatively reduced scar severity and improved ECM organization (Figures 2–4). In contrast, blank group scars exhibited thickened epidermal and dermal layers, irregular collagen fiber organization, and excessive ECM accumulation. Furthermore, the formation of new blood vessels in the H-CEFAE group showed a significant reduction over time, resulting in decreased blood supply, which is beneficial for suppressing scar tissue development. Although the L-CEFAE group demonstrated a greater quantity of new blood vessels compared to the H-CEFAE group, it also exhibited a tendency toward vascular inhibition (Figure 5). Quantification of COL I, COL III, and VEGF also supports these results (Figure 6).

## 4 Discussion

HS develop due to an aberrant wound-healing response characterized by excessive fibroblast proliferation, collagen deposition, and disrupted ECM remodeling. Central to this pathology is the aberrant behavior of COL I and COL III. Early in healing, COL III – a thinner, more flexible collagen variant – is overproduced, forming a provisional matrix that is later replaced by the thicker, sturdier COL I in healthy tissue. However, in HS, this transition is disrupted: COL III remains overly abundant initially, while COL I eventually dominates but is deposited in dense, parallel bundles rather than the basket-weave pattern of normal skin. VEGF promotes excessive microvascular proliferation, contributing to scar erythema and rigidity. Genetic predisposition and environmental factors (e.g., wound infection, delayed epithelialization) further amplify these pathways, culminating in raised, erythematous, and inelastic scars confined to the original wound boundaries. Unlike keloids, HS lack invasive growth but share overlapping molecular drivers.

Recent research advances in the treatment of HS using stem cells and exosomes have focused on harnessing their regenerative and anti-fibrotic properties to target the pathological mechanisms underlying scar formation [45]. MSCs, sourced from adipose tissue, bone marrow, or umbilical cord, are increasingly recognized for their ability to modulate inflammation, reduce fibroblast overactivity, and promote collagen remodeling. Studies highlight that MSCs inhibit HS progression by suppressing the TGF-β1/Smad pathway, a key driver of fibrosis, while enhancing the expression of anti-fibrotic factors like HGF and MMPs to degrade excess collagen. Innovations in delivery systems, such as 3D-printed scaffolds or injectable hydrogels, have improved MSC retention and localized therapeutic effects in scar tissue, addressing earlier challenges like poor cell survival. Exosomes, nanosized EVs derived from MSCs, have emerged as a safer, cell-free alternative, circumventing risks associated with whole-cell therapies. These vesicles are enriched with bioactive molecules, including miRNAs (e.g., miR-29a, miR-21, and miR-196a), proteins, and lipids, which regulate fibroblast proliferation, collagen synthesis, and inflammation. Preclinical models demonstrate that exosome treatment reduces scar thickness, improves collagen alignment, and normalizes ECM composition by silencing pro-fibrotic genes and promoting apoptosis in hyperactive fibroblasts. Advances in bioengineering, such as modifying exosomes to enhance targeting or loading them with specific anti-scarring miRNAs, have boosted their therapeutic precision. Techniques like exosome-laden microneedling patches or hybrid hydrogels enable sustained, controlled release into dense scar tissue, improving efficacy.

Despite their therapeutic potential, stem cell and exosome therapies for HS face significant challenges. Stem cells, particularly MSCs, struggle with low survival and engraftment rates in the harsh fibrotic scar microenvironment, where hypoxia and inflammation limit their effectiveness. Additionally, heterogeneity in MSCs sources and potency, along with risks of unintended differentiation or rare tumorigenic effects, raises safety and consistency concerns. Exosomes, while safer as cell-free alternatives, confront hurdles in standardization, as isolation methods yield variable purity and cargo composition. Their natural molecular cargo – miRNAs, proteins, and lipids – can include unintended pro-fibrotic elements, and engineering exosomes to enrich specific therapeutic molecules without compromising stability remains technically difficult. Both therapies face delivery challenges, as dense scar tissue impedes penetration, and current strategies (e.g., hydrogels, microneedling) only partially improve localized retention.

CEFAE is a cell-free biologic derived from processed adipose tissue, enriched with growth factors, cytokines, EVs, and signaling molecules. Its therapeutic potential for HS lies in its ability to target multiple pathological processes simultaneously. Angiogenesis is enhanced through VEGF and FGF, improving blood flow and reducing hypoxia-driven fibrosis. Simultaneously, CEFAE remodels the ECM by stimulating elastin and hyaluronic acid synthesis, which restores skin elasticity, while EV-mediated delivery of MMPs and miRNAs (e.g., miR-196a) degrades disorganized collagen and promotes healthy ECM alignment.

The results indicate that both low and high concentrations of CEFAE have a significant regulatory effect on collagen, particularly under high concentration conditions. Specifically, in the high concentration group, the content of COL III exhibits a decreasing trend, while the content of COL I gradually increases over time. CEFAE effectively rebalances collagen dynamics in HS by targeting both type 1 and COL III. It suppresses excessive COL I synthesis – associated with scar rigidity, while promoting the production of COL III, which enhances skin elasticity, through regenerative factors such as VEGF and FGF. We speculate that CEFAE upregulates matrix metalloproteinases (MMPs-1/3) to degrade overabundant COL I and ensures balanced degradation of COL III, maintaining a healthy collagen ratio simultaneously. This will be further validated in our future experiments. This dual modulation restores the collagen architecture to a natural, organized basket-weave pattern, improving scar pliability and tensile strength. Long-term, CEFAE sustains ECM homeostasis via bioactive molecules like adiponectin and miRNAs, which fine-tune collagen gene expression and prevent scar recurrence. By harmonizing collagen reduction and regeneration, CEFAE transforms HS into softer, thinner, and more functional tissue resembling healthy skin.

In comparison to the other cell and active factor therapies previously discussed, CEFAE presents several significant advantages. First, CEFAE circumvents the ethical challenges associated with MSCs. Currently, the use of allogeneic MSCs necessitates rigorous donor screening and informed consent, while the use of embryonically derived MSCs raises ethical concerns regarding the beginning of life. Moreover, traditional cell therapies often require *in vitro* expansion, which carries the risk of tumorigenesis. A notable advantage of CEFAE is that it does not involve the transplantation of live cells, thereby mitigating the risks of tumor formation and immune rejection. Additionally, CEFAE does not depend on the viability of donor cells, facilitating the establishment of a storage bank and eliminating the human leukocyte antigen matching issues commonly associated with MSCs. This study evaluated the safety and efficacy of rabbit autologous CEFAE, thereby establishing a foundational basis for its conceptual validation in human applications. Future research will concentrate on developing the production process for human-derived CEFAE and investigating its mechanisms for enhancing healing in chronic wound models. This acellular therapy model is anticipated to address the challenges associated with cell therapies, particularly in the realms of ethics, regulation, and industrialization.

However, challenges remain, including variability in CEFAE composition due to donor differences and processing methods, limited penetration into dense scars, and incomplete understanding of how individual components synergize. Compared to cell-based therapies, CEFAE avoids risks like tumorigenicity and offers a broader bioactive profile than isolated exosomes, but standardization and delivery optimization (e.g., combining with microneedling) are needed. Overall, CEFAE represents a promising, multi-target approach to scar treatment, though further clinical validation and mechanistic studies are essential to solidify its role in practice.

## 5 Conclusion

In conclusion, CEFAE demonstrates significant preventive and therapeutic efficacy in the rabbit ear HS model, effectively restoring homeostasis in collagen metabolism and inhibiting abnormal neovascularization associated with altered angiogenic regulators. These findings highlight the dual mechanisms through which CEFAE functions, particularly in the regulation of collagen remodeling and the angiogenesis process. This study supports the potential of CEFAE as a cell-free therapeutic intervention for HS, offering a promising approach for alleviating this condition. Future research will aim to further optimize the effective concentration of CEFAE.
